# Tailored Out-of-Oven Energy Efficient Manufacturing of High-Performance Composites with Two-Stage Self-Regulating Heating via a Double Positive Temperature Coefficient Effect

**DOI:** 10.1021/acsami.3c12901

**Published:** 2023-11-21

**Authors:** Xudan Yao, Yushen Wang, Thomas D. S. Thorn, Shanshan Huo, Dimitrios G. Papageorgiou, Yi Liu, Emiliano Bilotti, Han Zhang

**Affiliations:** † School of Engineering and Materials Science, Queen Mary University of London, London E1 4NS, U.K.; ‡ School of Aeronautics, Northwestern Polytechnical University, Xi’an 710072, China; § Department of Materials, Loughborough University, Loughborough LE11 3TU, U.K.

**Keywords:** sustainable manufacturing, conductive polymer composite, nanocomposites, graphene nanoplatelets, out-of-oven curing

## Abstract

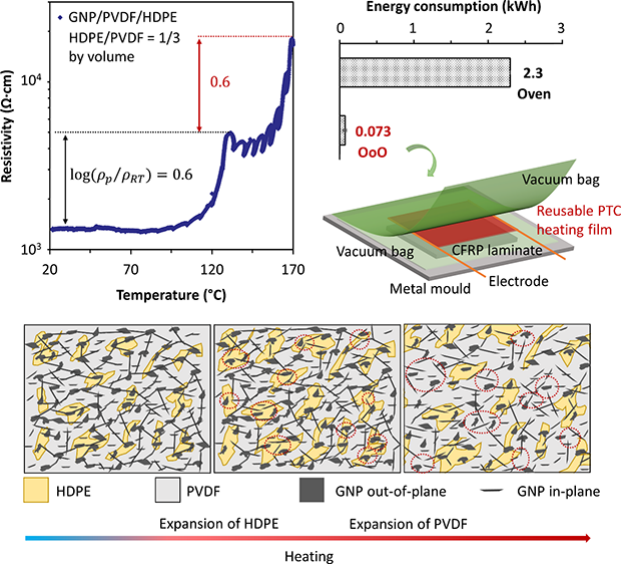

The needs for sustainable development and energy efficient manufacturing are crucial in the development of future composite materials. Out-of-oven (OoO) curing of fiber-reinforced composites based on smart conductive polymers reduces energy consumption and self-regulates the heating temperature with enhanced safety in manufacturing, presenting an excellent example of such energy efficient approaches. However, achieving the desired curing processes, especially for high-performance systems where two-stage curing is often required, remains a great challenge. In this study, a ternary system consisting of graphene nanoplatelets/HDPE/PVDF was developed, with a double positive temperature coefficient (PTC) effect achieved to fulfill stable self-regulating heating at two temperatures (120 and 150 °C). Systematic studies on both single and double PTC effects were performed, with morphological analysis to understand their pyroresistive behaviors. Compared to the oven curing process, up to 97% reduction in the energy consumption was achieved by the ternary system, while comparable thermal and mechanical properties were obtained in the carbon fiber/epoxy laminates. This work presents a new route to achieve OoO curing with two-stage self-regulating heating, which can be utilized in many high-performance composite applications.

## 1 Introduction

With the increasing concerns over the environmental impact of industrial processes, novel technologies and materials with high energy efficiency and low carbon footprints are increasingly in demand. Thanks to the high specific strength and stiffness, as well as good chemical resistance, fiber-reinforced plastics (FRPs) have been widely used as environmentally friendly lightweight solutions in diverse fields, such as aerospace, automotive, civil, energy, and sports. Unfortunately, the manufacturing of advanced composites using traditional methods, such as autoclave and oven-based curing processes, often leads to high energy consumption and size restrictions.

To overcome these limitations, out-of-oven (OoO) curing methods have gained much attention in recent years, ranging from microwave heating,^1−4^ induction heating,^4−6^ frontal polymerization,^7−9^ heated tooling,^10−12^ to resistive heating. In particular, resistive heating (or Joule heating), especially the use of a surface conductive layer such as carbon nanotubes or graphene, has been explored as one of the promising methods owing to its high compatibility with existing fiber/matrix systems, and ease of fabrication compared to integrally heated tooling. A wide range of resistive heating layers have been studied, such as carbon nanotube (CNT) film,^13−16^ graphene film,^17−20^ conductive wires,^21^ etc. However, the associated risks of overheating and burning from the excellent heating performance of many resistive heating systems should not be underestimated. The risks of malfunction or failure in the temperature controller, such as a proportional integral derivative (PID) controller, for the feedback loop in heated tooling also should not be overlooked, as they could lead to substantial heat-related hazards or production disruptions. The development of smart conductive heating layers that can self-regulate their heating temperatures presents a novel and exciting avenue for the sustainable manufacturing of composites.

Very recently, our group has conducted studies on the pyroresistive performance of conductive polymer composites (CPCs), leading to the development of reliable self-regulating heating performance which can be utilized for sustainable OoO curing with in situ temperature adjustment.^22−27^ By employing a single-step positive temperature coefficient (PTC) effect, which involves a sharp increase in the electrical resistance of the CPC due to the polymer matrix thermal expansion at the switching temperature (*T*_sw_), the heating can be self-regulated without overheating due to the disconnection of conductive pathways. When the temperature drops below *T*_sw_, the polymer shrinks back, reconnecting the conductive pathways and resuming the heating with the temperature under control.^25,27^ Compared to composites cured by traditional oven heating, OoO curing requires only a few percentages of energy consumption, which significantly contributes to the sustainable manufacturing of composite materials,^22,27^ especially considering the growing demand of advanced composites. The integrated CPC layer used in OoO curing also can be utilized as an embedded smart surface layer, providing additional functions ranging from structural health monitoring to deicing, toward a multifunctional lightweight composite structure.

However, the capability of self-regulate the heating at one switching temperature also poses a challenge for high-performance FRPs, whereas a two-stage curing process is often required with a post-curing step at an elevated temperature beyond the first stage. Unfortunately, the single PTC effect with an autonomous cutoff of conductive pathways cannot fulfill the needs of post-curing at a further elevated temperature. To solve this challenge, a double (two-step) PTC effect needs to be developed to fulfill the requirement. Different routes have been explored to achieve the double PTC effect over the last few years. Zhang et al. filled high-density polyethylene (HDPE) with Sb–Pb alloy and achieved double PTC effects owing to different melting points of the polymer matrix and alloy fillers.^28,29^ The introduction of a secondary filler, such as VO_2_ which has the phase-transformation characteristic of changing from semiconductor to metal at around 68 °C, contributed to the double PTC effect in graphite/HDPE composites as reported by Zhang and co-workers.^30,31^ Binary polymer matrix nanocomposites based on polymer blends with a two-stage thermal expansion hence two stages of resistance jump have also been explored.^32−34^ Feng et al.^34^ proposed the type I + M (Interface + Matrix) conductive pathway theory by combining carbon black (CB), polypropylene (PP), and ultrahigh molecular weight polyethylene (UHMWPE) via melt processing, and studied the effect of the PP/UHMWPE ratio on the PTC intensities of each stage. Wei et al.^33^ manufactured conductive CB/PP/UHMWPE composites via a different processing method: grinding and solution mixing, followed by hot compaction, with an ultralow percolation threshold achieved owing to the segregated structure, and a double PTC effect observed. Zhang et al.^32^ used carbon nanofiber (CNF), HDPE, and poly(vinylidene difluoride) (PVDF) with different matrix volume ratios, and only obtained double PTC at 1/4 ratio with a relatively low PTC intensity of the second step, explained by the “island-bridge” theory. The second PTC effect was attributed to the thermal expansion of PVDF which broke the CNF “bridges” between CNF/HDPE “islands”. As the separation of conducting pathways from this stage is much less than the first stage when HDPE expands and separates the network within the “islands”, the PTC intensity of the second stage was much lower than the first stage. In order to achieve a reliable two-step control on self-regulating heating, a sufficient PTC intensity is required for both stages.

To achieve a clear PTC effect with sufficiently high intensity, semicrystalline polymers are often favored, owing to their relatively large thermal expansions when approaching their melting temperatures of the crystalline phase.^23,25^ Hence, two semicrystalline polymers, HDPE and PVDF, with distinct melting points (∼130 and ∼175 °C) are utilized in this work to fulfill the two-step curing cycle requirements. Apart from the PTC effect, Joule heating is another function that is vital for OoO curing, which ideally needs both high PTC intensity for smart switching and appropriate resistance for Joule heating. Regarding the conductive filler selection, Liu et al.^23^ compared 0D (silver-coated glass spheres (AgS)), 1D (CNT), and 2D (graphene nanoplatelet (GNP)) fillers. It was found that the specimens consisted of 0D filler tend to have the highest PTC intensity with the lowest resistivity at the highest loading due to its least number of contact points and lowest specific surface area, the 1D CNT filler shows the lowest PTC intensity owing to its “robust” conductive networks with many entanglements that are the least likely to be broken, while the 2D GNP behaves intermediately in both PTC intensity and loading for percolation threshold.

In this study, CPC with double PTC effect is fabricated and utilized as a surface layer to cure the high-performance carbon fiber (CF)/epoxy laminates, with a two-stage curing cycle. A systematic characterization of the composite laminates cured by OoO and the traditional oven method has been performed, comparing their thermomechanical, morphological, and mechanical performance, while the energy consumption between the two methods was also measured and compared. The results indicate that OoO-cured CF/epoxy laminates possess equivalent properties to the traditional oven-cured specimens, with a significantly reduced energy consumption toward the sustainable development of this field.

## 2 Experimental Section

### 2.1 Materials

GNPs (XG sciences, grade M25, USA) were used as the conductive fillers in this work, which have an average particle diameter of 25 μm, thickness of 6–8 nm (∼20 layers of graphene), and density (ρ_g_) of 2.2 g cm^–3^ according to the manufacturing datasheet. PVDF with particle size <300 μm, density of 1.78 g cm^–3^ (Solef 1015, Belgium) and HDPE with density of 0.952 g cm^–3^ (RIGIDEX, HD5218EA, UK) were used as the polymer matrix, with the melting temperatures (*T*_m_) at around 175 and 130 °C, respectively, with the aim of matching the PTC switching temperatures (which are often slightly below the *T*_m_) to the curing cycle of the epoxy resin system (Araldite LY 1564, Aradur 2954, USA). Stitched CF fabric was obtained from Hexcel (HiMax FCIM151, 303 gsm, −45/+45, USA). A 300 gsm nonwoven heavyweight polyester felt fabric (BR180) was bought from Easy Composites Ltd. (UK) and used as the thermal insulation layers during the OoO curing process. All polymer matrices were dried in the oven at 80 °C overnight before processing. Strips of 3 mm-wide copper tapes combined with 0.056 mm-thick copper wires and meshes were used as the electrical buses to connect the samples to a power supply.

### 2.2 PTC Nanocomposite Preparation

**Figure 1:**
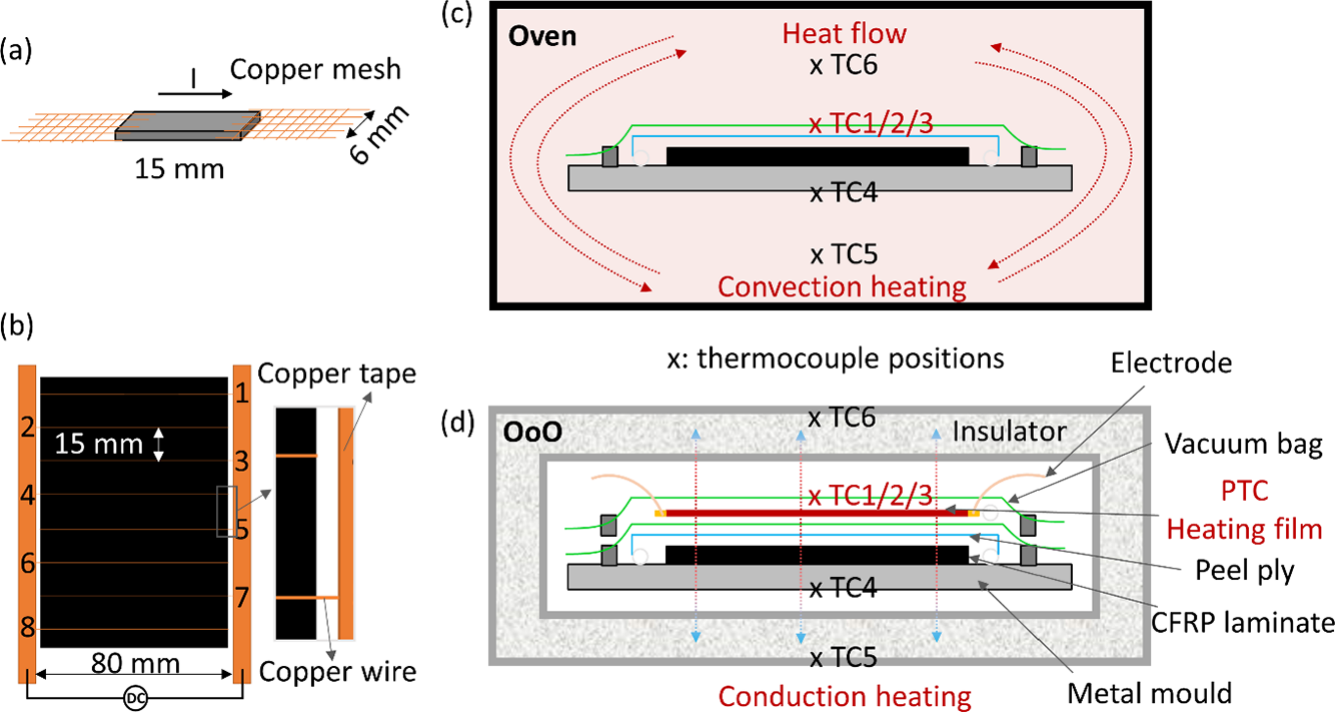
Schematic illustrations of nanocomposite fabrication: (a) PTC test specimens with embedded copper mesh as electrodes; (b) Joule heating films with parallel copper wire electrodes embedded via compression molding; (c) oven and (d) OoO with thermal couples monitoring the temperatures at various locations during the curing process. For OoO curing, the CPC film was sandwiched between two vacuum bagging layers, with a DC power supply connected for Joule heating. Thermal insulation was applied with temperatures monitored at different locations.

A DSM X’plore 15 micro-compounder (the Netherlands) was used for mixing 24 wt % GNP into polymer matrices. The compounding was carried out at 240 and 200 °C for PVDF and HDPE, respectively, at a speed of 50 rpm under argon for 5 min. The extruded filaments were cut into pellets by a Dr. Collin Strand Pelletizer Type CSG 171 (Germany). The pellets were then hot pressed into films and sheets using a Dr. Collin P300E (Germany) at 200 °C under 240 bar. For GNP/HDPE/PVDF trinary system, GNP/HDPE and GNP/PVDF pellets with a HDPE/PVDF volume ratio of 1/3 were mixed for 3 min at 240 °C by the micro-compounder. For PTC test specimens, copper meshes were embedded in the nanocomposite during the compression molding as shown in Figure 1a. To fabricate the Joule heating films based on developed CPC, parallel copper wires with an interval of 15 mm were embedded into the film via compression molding (Figure 1b), with a final film thickness of around 200 μm.

### 2.3 CF/Epoxy Laminate Manufacturing

CF/epoxy laminates (100 mm × 100 mm) consist of 8-ply CF with a layup of [0/90]_2s_ were manufactured by a vacuum-assisted resin infusion (VARI). After infusion, the whole assembly was placed into an oven for curing, as shown in Figure 1c. In comparison, considering the recyclability, reusability, and cost effectiveness of OoO curing method, the heating film was placed on top of the whole VARI assembly, with another vacuum bag sealed and vacuumed to ensure the contact between the smart heating film and the CF/epoxy panel (Figure 1d). The curing cycle was based on the suggested profiles from the supplier, at 120 °C for 0.5 h followed by 150 °C for 2 h. Three CF/epoxy laminates were made from each curing methods.

In order to reduce the heat loss, 8 layers of polyester felt fabric were applied as the thermal insulation layers to wrap the whole assembly after infusion. Three thermocouples (TC1/2/3) were placed at three different positions on the surface of the assembly to monitor the heating behavior. One thermocouple (TC4) was adhered to the bottom surface of the stainless-steel mold. For OoO curing, two additional thermocouples (TC5 and TC6) were placed between the seventh and eighth layers of the polyester insulation fabric (Figure 1d) to monitor the heat loss. While for oven curing, TC5/6 were used to monitor the air temperature within the oven.

### 2.4 Characterization

A FEI Inspect F field emission scanning electron microscopy (SEM) was used to observe the morphology of GNPs and polymers, as well as cryo-fractured cross sections of the composites. Thermogravimetric analysis (TGA 5500, TA Instruments) was used to check the final GNP loadings in the CPCs, with the samples heated up from room temperature (RT) to 800 °C at 10 °C/min under nitrogen atmosphere. Glass transition temperature (*T*_g_) of cured CF/epoxy laminates was evaluated by differential scanning calorimetry (DSC25, TA Instruments), with temperature ramped from 40 to 200 °C at a heating rate of 10 °C/min under nitrogen atmosphere. Dynamic mechanical analysis (DMA Q800, TA Instruments) was performed in accordance with ASTM D7028, with the CF/epoxy specimens dimension of 56 mm × 12 mm × 1.5 mm under three-point bending mode, from RT to 200 °C at 5 °C/min heating rate, frequency of 1 Hz, strain of 0.1% and preset force of 0.1 N, under air environments.

The pyroresistive behavior of CPC smart layers was characterized by measuring the electrical resistance (Agilent 34410A 6 1/2 Digit Multimeter) of the PTC specimens under various temperatures (monitored by K-type thermocouples and a TC-08 Pico logger) simultaneously. To examine the Joule heating performance of the film, a GE EPS 301 power supply (voltage range: 5–300 V DC; current range: 10–400 mA) was used with different voltages applied. K-type thermocouples accompanied by a TC-08 thermocouple data logger from Pico Technology were used to record the temperatures of various positions on the film surfaces.

Flexural properties of CF/epoxy laminates were tested by Instron 5967 under three-point bending mode according to ASTM D7264, with the sample dimension of 60 mm × 12 mm × 1.5 mm and support span of 48 mm (span-to-thickness ratio of 32:1). The crosshead movement was set at a rate of 1.0 mm/min, with a 1kN load cell. The flexural chord modulus of elasticity was calculated using the elastic linear deformation range (e.g., strain starts from 0.001 and ends at 0.003). For each method, nine specimens in total, from three different panels, were tested to ensure the repeatability of the results.

## 3 Results and Discussion

### 3.1 Morphological, Pyroresistive, and Self-Regulating Heating Properties of Single PTC Nanocomposites

**Figure 2:**
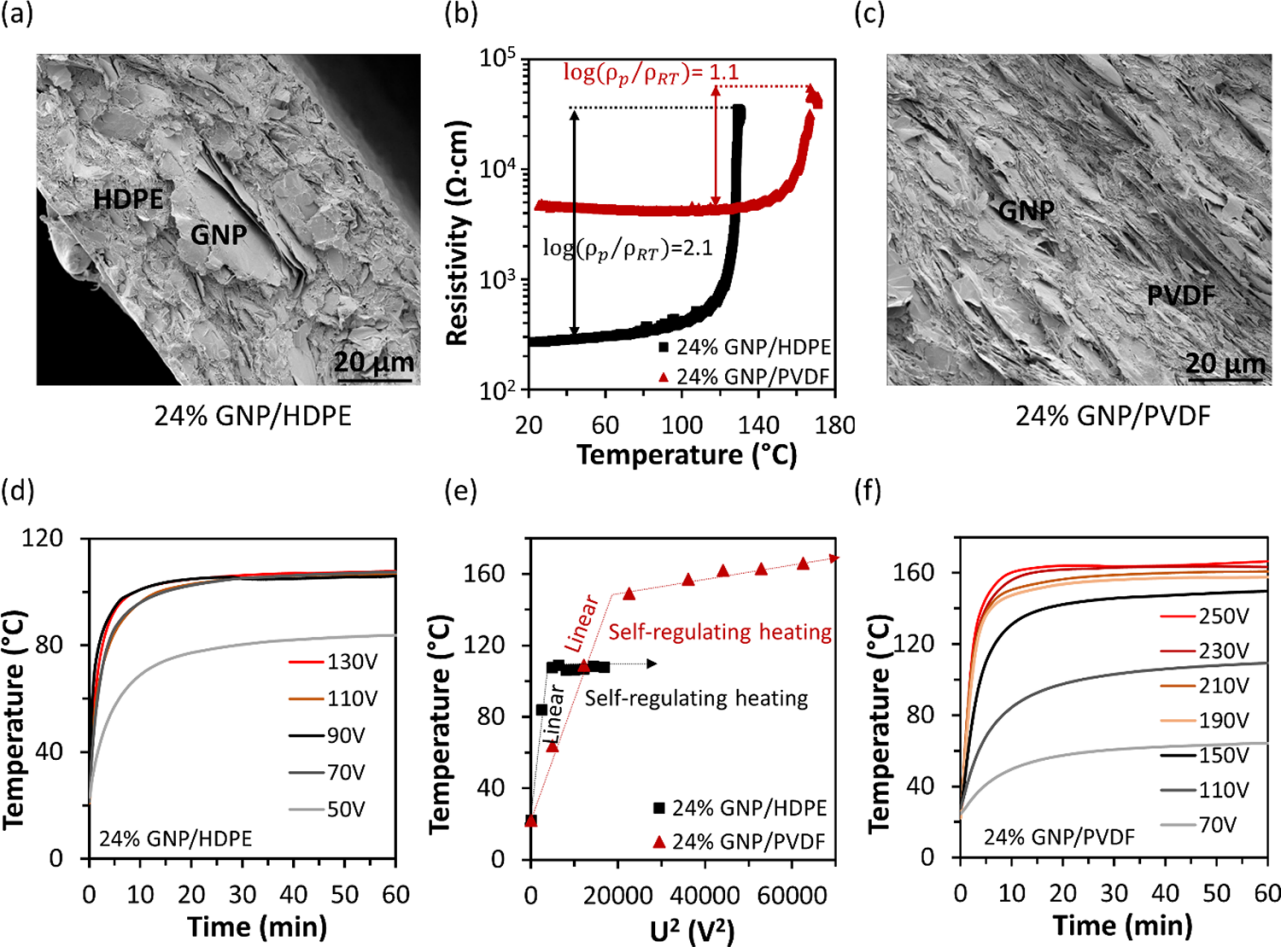
SEM images of cryo-fractured cross sections of 24 wt % GNP in (a) HDPE and (c) PVDF, with GNPs distributed with sufficient connecting pathways inside; (b) pyroresistive behavior of GNP/HDPE and GNP/PVDF nanocomposites, with PTC intensity of 2.1 (owing to the relatively high coefficient of thermal expansion of HDPE) and 1.1, and switching temperatures at around 120 and 160 °C respectively. Joule heating behavior of (d) GNP/HDPE and (f) GNP/PVDF composites under various applied voltage. Both composites achieved self-regulating heating, with the temperatures stabilized at around 105 and 155 °C, respectively, independent of further increased voltages, which is promising for safe manufacturing without the risk of overheating. (e) *U*^2^ – *T* curves of the nanocomposites, which are linear before the switching temperatures and stabilized at constants (∼105 and ∼155 °C) afterward, confirming the self-regulating heating.

Figure 2a,c shows the morphology of the cryo-fractured cross-sectional views of 24 wt % GNP/HDPE and 24 wt % GNP/PVDF nanocomposites, respectively. Continuous distribution of GNPs with sufficiently connected pathways between fillers can be found in both matrices, which can be attributed to the relatively high filler contents alongside appropriate shear force applied during the melt-mixing process. An in-plane aligned distribution of GNPs has been observed, and its formation is believed to be due to the compression molding process.

Pyroresistive behavior of the nanocomposites was studied by monitoring their electrical resistivity changes over increased temperature. As shown in Figure 2b, both nanocomposites show a clear single-step PTC effect, with switching temperatures at around 120 and 160 °C, which are attributed to the thermal expansion of the crystalline phase when approaching the melting point of each matrix. The PTC intensity, as calculated in eq 1 below, is 2.1 for the HDPE system and 1.1 for the PVDF system, in agreement with their coefficient of thermal expansion (CTE) values:
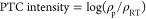
1where ρ_p_ and ρ_RT_ are the peak and RT resistivity, respectively. The PTC intensity difference between 24 wt % GNP/HDPE and 24 wt % GNP/PVDF composites is mainly attributed to their CTE difference, where the CTE values of HDPE and PVDF are at the range of 200–250 and 110–130, respectively (10^–6^ K^–1^ at 20 °C). A higher CTE value can enable a larger volume expansion of the matrix, hence breaking more conducting pathways and leading to a higher PTC intensity. It is also worth noting that, with a constant filler loading, the matrix viscosity will also influence the initial electrical conductivities due to the filler movements and reagglomerations during the melt processing, contributing to the calculation of PTC intensity.

**Figure 3:**
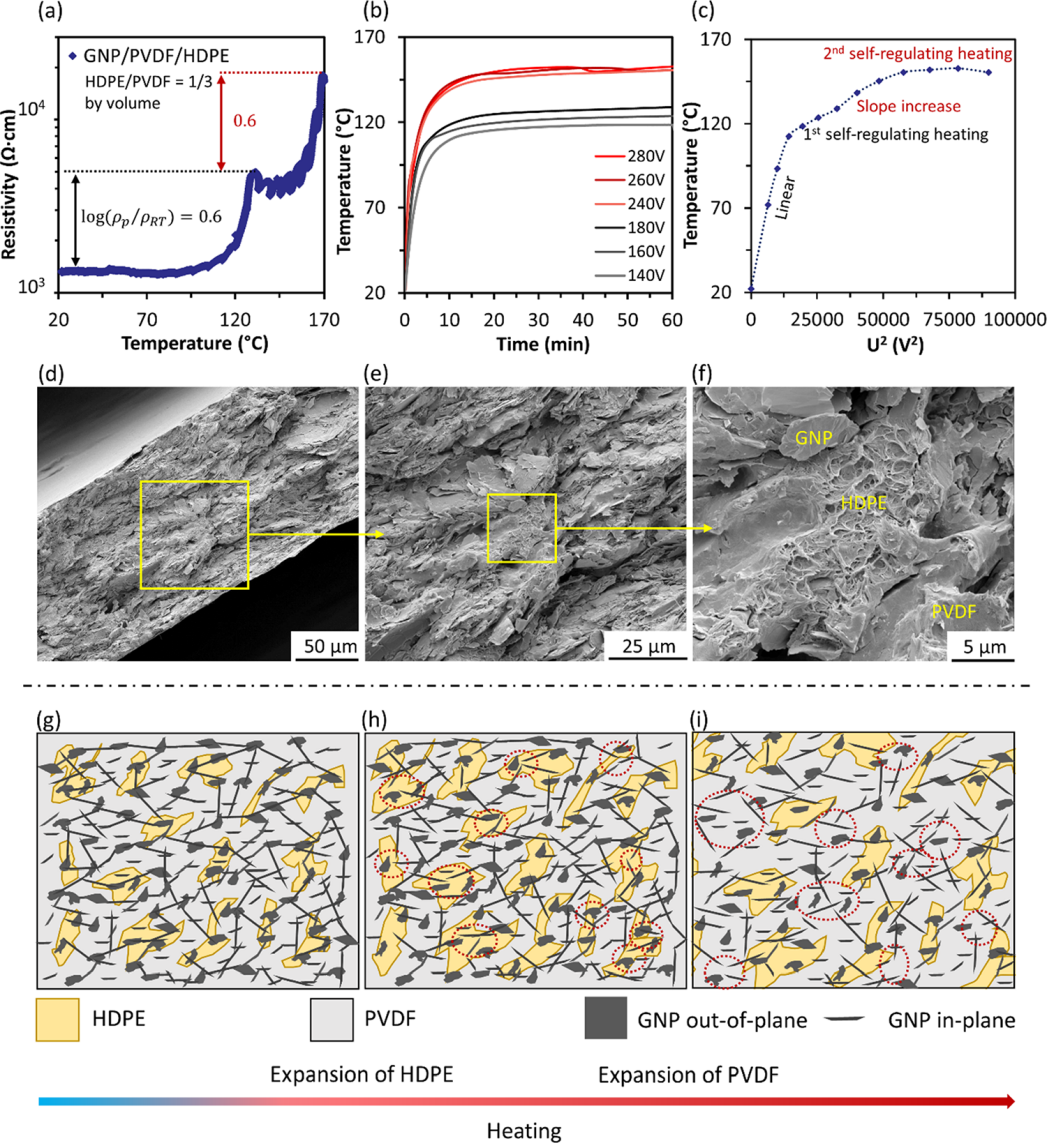
GNP/HDPE/PVDF ternary nanocomposites with 24 wt % GNP loadings (volume ratio of HDPE and PVDF is 1:3): (a) pyroresistive properties with a clear double PTC effect; (b) Joule heating behavior with a two-stage self-regulating heating in accordance with two PTC switching temperatures; (c) *U*^2^–*T* curve with two clear slope changes at the self-regulating temperatures (∼120 and ∼150 °C); (d–f) cross-sectional views of ternary nanocomposites, showing a continuous phase of PVDF with GNPs dispersed in both PVDF and HDPE phases, as well as their interfaces. (g–i) Schematic illustrations of the evolution of conductive networks in the ternary nanocomposites upon heating, with the thermal expansion of HDPE and PVDF.

A relatively high PTC intensity with a clear and sharp resistance jump can contribute to the self-regulating heating performance of the nanocomposites. Both 24 wt % GNP/HDPE and 24 wt % GNP/PVDF composite films showed reliable self-regulating heating, as shown in Figures 2 and 3f. For GNP/HDPE nanocomposites, with the increasing voltage applied (e.g., 50–70 V), the peak temperature increased from 80 to 105 °C due to the increased power input. However, with the voltage further increased from 70 to 90, 110, or even 130 V, the peak temperature kept constant at around 105 °C (Figure 2d), achieving self-regulating heating. This is attributed to the PTC effect of the 24 wt % GNP/HDPE nanocomposite. When temperature approaches *T*_sw_, the resistance of composites increases dramatically, compensating the further increased voltage inputs (*U*^2^/*R*), thus leading to stable output powers and preventing overheating. Similarly, for GNP/PVDF nanocomposites, a stable self-regulating heating has been observed at 155 °C (Figure 2f), with the applied voltage increased from 190 to 250 V.

During the Joule heating process, thermal insulating materials (polyester felt fabric) were used to cover the samples to prevent the major heat loss, convection.^35^ In this case, energy generated by resistive heating contributes to heat up the composite accompanied by heat loss via heat conduction and radiation, following the equations below:
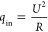
2
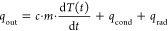
3

According to the law of conservation of energy:
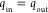
4
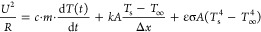
5where *U* is the applied voltage; *t* is the time; *R*, *T*, *c*, and *m* are the resistance, temperature, specific heat capacity, and mass of the samples; *A* is the heating area; *T*_s_ and *T*_∞_ are the sample surface and environmental temperature, respectively; Δ*x* is the distance between sample surface and environment; *k*, ε, and σ are the thermal conductivity of the insulating material, emissivity, and Stefan–Boltzmann constant, respectively.

Heat radiation is proportional to the fourth power of the absolute temperature, which will become the dominate part when the temperature becomes extremely high. In this work, temperatures are relatively modest, and thus, the major heat loss is through conduction. Thus, eq 5 could be simplified as
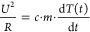
6

As a consequence, for both GNP/HDPE and GNP/PVDF nanocomposites before the switching temperatures (*T*_sw_), the relationship between *U*^2^ and *T* was linear, as shown in Figure 2e. After *T*_sw_, temperatures of both systems become stabilized without further increases (∼105 and ∼155 °C), indicating the safe self-regulating heating achieved owing to the resistance jump.

### 3.2 Double PTC Nanocomposites with Two-Stage Self-Regulating Heating

In order to achieve a two-step OoO curing process for high-performance FRP composites, a self-regulating heating film with a double PTC effect is of necessity. We developed a ternary nanocomposite system by melt-mixing the GNP/HDPE and GNP/PVDF at desired volume ratios, maintaining the conductive filler concentrations of 24 wt %. Considering the difference in melting temperature between HDPE and PVDF, hence the self-regulating temperature (∼105 and ∼155 °C) as observed in Figure 2, a volume ratio of 1/3 has been chosen to achieve a continuous PVDF phase with higher switching temperature. This is to ensure the continuity of the conductive pathways as well as the matrix at elevated temperatures, especially after the first self-regulating temperature but before the second switching temperature. It is also worth noting that due to the surface tension of polymer matrix, GNPs tend to stay in HDPE rather than PVDF.^23^ Therefore, a lower HDPE volume can also avoid excessive migration of GNPs from PVDF to the HDPE phase. The chosen volume ratio is also expected to achieve balanced PTC intensities between two switching temperatures, due to their different coefficient of thermal expansion and PTC intensity values.

As shown in Figure 3a, a double PTC effect has been achieved in our GNP/HDPE/PVDF ternary nanocomposites. Upon heating, the resistance of the specimen remained stable from RT until around 105 °C, with a clear jump around 120 °C. With the further increased temperature, the resistance fluctuated slightly at the same level, until a further clear jump at around 160 °C. The temperatures of these two resistance jumps are in alignment with the switching temperatures of HDPE and PVDF phases, while the calculated PTC intensity for each stage is around 0.6.

The Joule heating performance of the specimen has been examined by applying voltages at different levels, with the temperature monitored and recorded simultaneously (Figure 3b). With the applied voltage increased from 140 to 180 V, an increased heating rate has been observed due to the higher power input, while the temperature reached around 120 °C and self-regulated at that temperature without further increases. This is attributed to the thermal expansion of HDPE phase at this temperature, in consistent with first PTC switching observed. With the applied voltage further increased to a much higher level (240 V), the temperature of the specimen can overcome the first regulating stage of HDPE, reaching about 150 °C and stabilized without further increase, even at a further increased voltage up to 280 V. This self-regulating phenomenon at 150 °C can be attributed to the thermal expansion of PVDF phase, pushing the remained conductive pathways apart hence to restrict any further heating. These self-regulating temperatures can be tailored by changing the polymer matrix used in the nanocomposite films, aligning with the required curing temperatures of the epoxy resins. It is worth noting that the relatively high voltage applied in this work is partially due to the limited current through the specimen (current range of 10–400 mA from the power source), which can be tuned by adjusting the film resistance between electrodes or using a higher current if needed.

As mentioned earlier in eq 6, a linear relationship between the square of applied voltage (*U*^2^) and temperature (*T*) can be expected for Joule heating at current temperature range. In Figure 3c, a linear relationship was observed at the beginning of the curve, until the temperature reaching 120 °C. A clearly reduced slope of the curve can be found at this temperature, which is due to the suddenly increased resistance of the nanocomposites at their first switching temperature (HDPE phase volume expansion). With the continued voltage square increase, the slope of the curve increased again with an increase in temperature, based on the remained conductive pathways within the PVDF phase, until the temperature reached around 150 °C where the thermal expansion of continuous PVDF phase was triggered, leading to a further resistance jump hence a self-regulated heating at this temperature. Morphological structures of current GNP/HDPE/PVDF ternary nanocomposites in Figure 3d–i also confirmed the continuous phase of PVDF, ensuring a connected pathway for Joule heating after first self-regulating heating stage while providing a safety current cut off at the second self-regulating temperature.

In order to check the thermal behavior and final GNP loadings of GNP/HDPE, GNP/PVDF, and GNP/HDPE/PVDF, TGA was performed from RT to 800 °C at 10 °C/min, under nitrogen atmosphere, with the results summarized in Figure S1 and Table S1. It indicates that GNPs (all at around 20 wt %) contributed to increased decomposition temperatures owing to its high thermal stability and restriction effect, which is consistent with the literature.^36,37^ In addition, the ternary nanocomposites resulted in improved thermal stability, with a slightly higher (∼20 °C) initial decomposition temperature (487.9 °C) compared with the GNP/HDPE (468.2 °C). As the HDPE was trapped by surrounding PVDF (Figure 3h), thermal expansion at the first switching temperature was also constrained; hence, a reduced separation of conductive network until a higher temperature in GNP/HDPE/PVDF was reached (120 °C), compared with GNP/HDPE single matrix system (105 °C). In contrast, the second switching temperature dropped slightly from 155 °C (GNP/PVDF) to 150 °C (GNP/HDPE/PVDF), attributed to the synergy thermal expansion of PVDF and HDPE (Figure 3i), which triggered an earlier cutoff of the conductive pathways.

### 3.3 Oven vs OoO Curing for High-Performance CF/Epoxy Laminates

After successfully achieved the two-stage self-regulating heating based on developed GNP/HDPE/PVDF ternary nanocomposites, the heating film was fabricated and used for OoO curing of CF/epoxy laminates with two-stage curing cycles. In comparison, traditional oven was also used to cure the laminates with the same curing cycle, while the heating profiles, the mechanical and thermomechanical properties of cured laminates, as well as their energy consumption, have been compared between two curing methods.

#### 3.3.1 Heating Performance for the Curing Cycle

**Figure 4:**
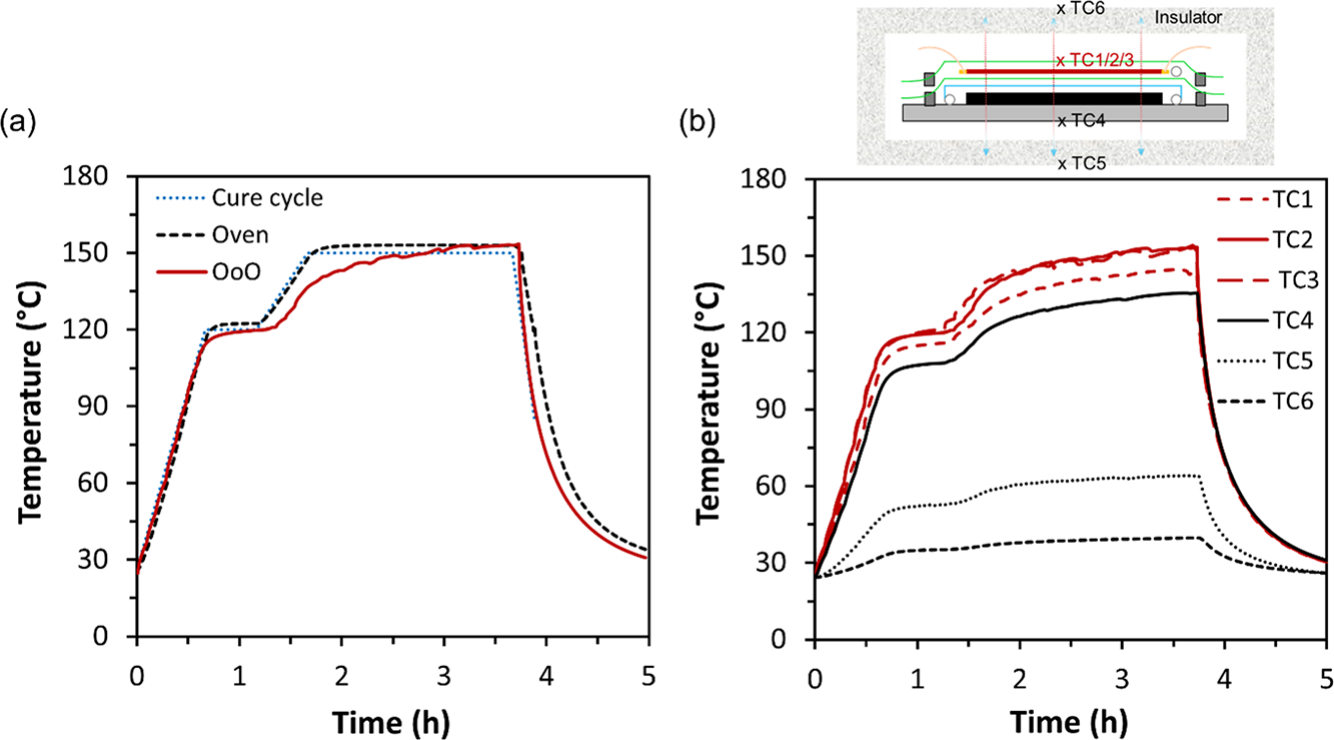
(a) Comparison of heating profile between oven and OoO curing methods, both based on reading from TC2, the central thermocouple on the surface of the whole assembly. (b) Temperature profile of the OoO curing assembly, with six thermocouples embedded at different locations: TC1/2/3 at three different positions on the surface of the heating film; TC4 was adhered to the bottom of the steel mold; TC5 and 6 were placed between the 7th and 8th layers of the insulating fabric.

Figure 4a compares the heating profiles between the oven and OoO curing methods, with the target cycles (dash line) of the first stage at 120 °C for 30 min and the second stage at 150 °C for 2 h. Both methods achieved the first curing stage at RT, while the OoO shows a slightly lower temperature than 120 °C. It is worth noting that this temperature for the OoO method was collected from TC2, the central thermocouple attached to the outer surface of the heating film. After the first curing stage, the temperature has been increased further to 150 °C for another 2 h before cooling down to RT. Although the heating rate of OoO method was slightly lower than the oven method for the post-curing stage (due to the limit of 300 V that could be applied from the power supply in this work), the temperature was continuously increasing until 150 °C and stabilized at that temperature. Clearly, with the laminates as well as the metal substrate underneath, a large amount of energy is required to raise the temperature, especially at the higher temperature range. It is believed with a higher voltage applied, the heating rate can be optimized for the system.

To further understand the heating performance of developed OoO method, the temperature profiles of all six thermocouples have been presented in Figure 4b. It can be seen that all three thermocouples on the heating film surface (TC1–3 in red) show a similar profile, with one of the lines slightly below the others, especially after the first curing stage and during the entire second stage, possibly due to the locations of parallel electrodes and subsequent variation in resistance. This also explains the slightly slower ramping rate of the OoO in comparison with oven method in Figure 4a. Decent thermal insulation has been observed for both side of the assembly (TC5 and 6), although a slightly elevated temperature can be seen from TC5 on the metal mold side, which can be attributed to the relatively compacted thermal insulation layers due to the gravity of metal mold.

It is also worth noting that the temperature of the steel mold was slightly lower (∼20 °C) than the film temperature, throughout the entire curing process. This is believed due to the relatively low power density of the heating film while the relatively large thermal mass (*C*_th_) of the metal plates.
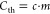
7
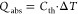
8where *c*, 502.416 J/(kg K), m, and Δ*T* represent the heat capacity, mass, and temperature difference of the steel mold, respectively. The calculated thermal mass of the mold is 866.2 J/K and the energy absorbed by the mold through the entire curing cycle would be 95.9 kJ. Therefore, it can be expected that with an alternative mold of less heat capacity, the heating performance could be further improved with reduced energy absorption from the mold.

#### 3.3.2 Performance of CF/Epoxy Laminates Cured by Both Methods

Regardless of the curing methods employed, it is essential to ensure the performance of the cured laminates remains unchanged. Both the mechanical and thermomechanical performances of the cured laminates have been examined and compared for two methods.

**Figure 5:**
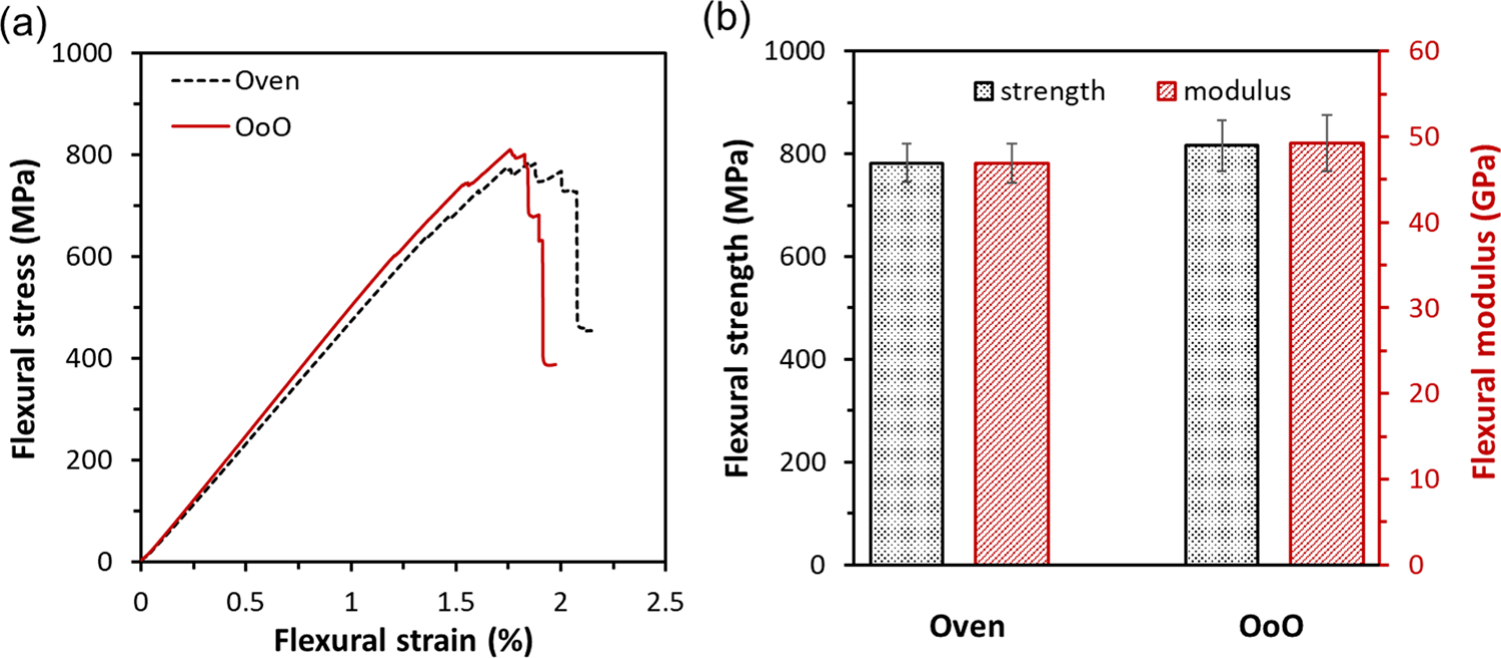
(a) Representative stress–strain curves and (b) flexural strength and modulus of CF/epoxy laminates, showing similar mechanical performance between laminates cured by oven and OoO methods.

Flexural properties of cured CF/epoxy laminates were characterized by three-point bending tests, with representative stress–strain curves, average flexural strength, and modulus summarized in Figure 5. All samples showed comparable flexural properties, without any obvious difference in their flexural properties. Additionally, the fiber volume fractions (*V*_f_) of oven and OoO-cured CF/epoxy laminates were calculated (details in SI) with the average values of 45.0 and 46.6%, respectively. The slightly higher fiber volume fraction of the OoO-cured CF/epoxy laminates led to slightly higher strength (4.3%) and modulus (4.9%), which could be attributed to the second vacuum bagging procedure of attaching the heating film. DSC and DMA analysis were utilized to evaluate the thermal and thermomechanical performances of both oven and OoO-cured laminates, with the results summarized in Figure S2 and Table S2. In short, both methods gave similar *T*_g_ and storage modulus at RT (48 and 50 GPa for oven and OoO-cured laminates). These results are consistent with flexural properties. Compared with traditional oven heating, a comparable result has been obtained from the OoO method, confirming its potential as an alternative curing method for composite laminates.

#### 3.3.3 Energy Consumption

**Figure 6:**
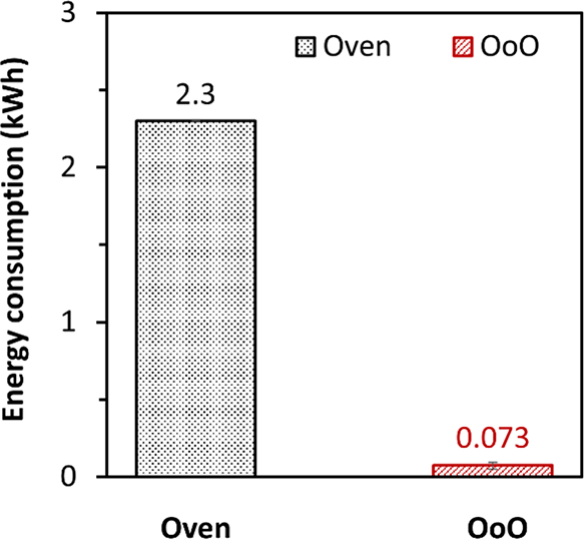
Comparison of energy consumption between oven and OoO method, indicating a significant energy reduction (97%) from the OoO curing method.

To compare the energy efficiency between oven and OoO method, real-time power consumption was recorded by a power meter and calculated for the entire curing process. Total energy consumptions of both methods, with three repeats for each method, are summarized and compared in Figure 6. Compared with traditional oven curing (2.3 kWh from each repeat), only 3% of energy was consumed from the OoO curing method (0.073 kWh), showing a significantly reduced energy consumption. This is believed due to the conduction heating from the heating film attached to the surface of CF/epoxy laminates, alongside thermal insulation, ensuring a highly efficient heat transfer from the Joule heating layer to the composite laminate. In contrast, a traditional oven needs to heat up the air in the oven first before the heat can be transferred to the laminates, with most energy consumed on heating the oven container. The power density of 0.24 and 0.30 W/cm^2^ were calculated for the heating film (84 cm^2^) at two steady-state curing stages, 120 and 150 °C, respectively, which are relatively low compared to the literature with similar temperatures.^13,15,18,38,39^ It is also worth noting that the amount of energy absorbed by the stainless-steel mold throughout the curing was calculated to be 95.9 kJ, which was about 36% of the overall energy consumption. This could be optimized by using a mold with lower thermal mass, hence to further increase the energy efficiency of the manufacturing process.

## 4 Conclusions

This work developed a tailored OoO curing method for sustainable manufacturing of high-performance CF/epoxy laminates, via a two-stage self-regulating heating based on the double PTC effect. Systematic studies on both single and double PTC effects were performed, with morphological analysis to understand their pyroresistive behaviors. Single PTC intensity of 2.1 and 1.1 was achieved at 120 and 160 °C, from GNP/HDPE and GNP/PVDF nanocomposites, respectively, while a double PTC with an intensity of 0.6 for both stages was successfully achieved from developed GNP/HDPE/PVDF ternary nanocomposites after turning the morphology and nanofiller network. Subsequently, a two-stage self-regulating heating has been achieved based on ternary nanocomposites, with heating temperatures of around 120 and 150 °C which corresponds to the curing temperatures of epoxy resins. The relationship between applied voltage and temperature was also characterized and compared for the developed system.

The tailored ternary nanocomposites with double PTC effect were utilized as an energy efficient and safe manufacturing method for high-performance CF/epoxy laminates’ out-of-oven (OoO) curing, fulfilling both curing and post-curing temperatures with self-regulating heating. Compared with traditional oven heating, a similar heating profile was achieved from the OoO method, leading to comparable mechanical and thermomechanical properties of the laminates cured by both methods. In addition, only 3% of energy consumption was used from the OoO method, indicating a significantly enhanced energy efficiency from the developed curing method based on nanocomposite. A relatively low power density in the range of 0.2–0.3 W/cm^2^ was achieved. With many favored features ranging from significantly reduced energy consumption, enhanced safety via self-regulating heating, and out-of-oven nature without size limitations, this work provides a promising route for sustainable manufacturing of high-performance composites.

## Supporting Information Available

TGA analysis of GNP nanocomposites; DSC and DMA results and thermal and thermomechanical performance of both oven and OoO-cured CFRPs; and fiber volume fraction calculation of cured CFRPs (PDF)
