# Supplementary material for: Tailored Out-of-Oven Energy Efficient Manufacturing of High-Performance Composites with Two-Stage Self-Regulating Heating via a Double Positive Temperature Coefficient Effect
Source: ACS Appl Mater Interfaces. 2023 Nov 21;15(48):56265–74. doi: 10.1021/acsami.3c12901 (PMC10711706; doi:10.1021/acsami.3c12901)
Supplement: Supplementary file 1 — am3c12901_si_001.pdf [file am3c12901_si_001.pdf]

Tailored out-of-oven energy efficient manufacturing of high performance composites with two-stage self-regulating heating via double positive temperature coefficient effect

*Xudan Yao [a,b,**], Yushen Wang [a], Thomas D. S. Thorn [a], Shanshan Huo [a], Dimitrios G. Papageorgiou [a], Yi Liu [c], Emiliano Bilotti [a], Han Zhang [a,*]*

[a] School of Engineering and Materials Science, Queen Mary University of London, London E1 4NS, UK

[b] School of Aeronautics, Northwestern Polytechnical University, Xi'an 710072, China

[c] Department of Materials, Loughborough University, Loughborough LE11 3TU, UK

* Corresponding author: han.zhang@qmul.ac.uk   x.yao@nwpu.edu.cn

Supporting Information

TGA analysis and final GNP loading of nanocomposites

In order to check the thermal behaviour and final GNP loadings, TGA was performed from RT to 800 °C at 10 °C/min, under nitrogen atmosphere, with the results summarized in Fig. S1 and Table S1. Final GNP loadings of GNP/HDPE, GNP/PVDF and GNP/HDPE/PVDF were at ~20 wt. %. Onset temperatures (T_{onset}), which represent the initial decomposition, of pure HDPE and PVDF were at 431.2 °C and 464.5 °C. The addition of 24 wt. % GNP increased the T_{onset} of HDPE and PVDF to 468.2 °C and 475.4 °C respectively, indicating an improved thermal stability for both cases, particularly for HDPE. Similarly, temperatures of maximum rate of degradation (T_{max}) also increased with the addition of GNP, as shown in Table S1. This

increment is attributed to the high thermal stability of GNP and its restriction effect, which is consistent with literatures [1,2]. As a result of the thermal stability improvement, 24 wt. % GNP/HDPE and 24 wt. % GNP/PVDF have similar T_{onset} and T_{max}, and thus the decomposition of HDPE and PVDF could not be differentiated by TGA analysis in the GNP/HDPE/PVDF trinary system. In addition, combination of GNP/HDPE and GNP/PVDF, namely the GNP/HDPE/PVDF, showed the highest T_{onset} and T_{max}.

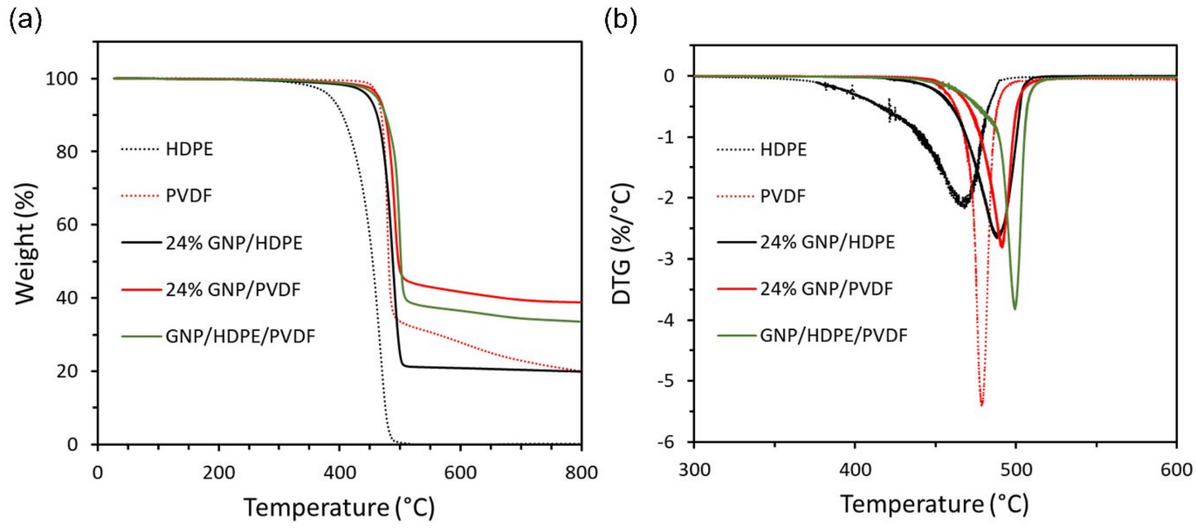

Fig. S1. (a) TGA and (b) DTG curves of pure HDPE, PVDF, 24 wt.% GNP/HDPE, 24 wt.% GNP/PVDF and GNP/HDPE/PVDF (HDPE/PVDF: 1/3 by volume).

Table S1. Final weight from TGA and the corresponding estimation of GNP loadings, as well as the temperature of onset and maximum rate of degradation.

	Residual weight (%)	GNP loading (%)	T_{onset} (°C)	T_{max} (°C)
HDPE	0.1±0.1		431.2±3.7	468.5±0.8

PVDF	20.3±3.6		464.5±10.4	477.6±2.7
24% GNP/HDPE	20.4±1.5	20.3	468.2±3.3	488.2±3.1
24% GNP/PVDF	37.8±1.4	21.0	475.4±1.9	490.2±2.1
GNP/HDPE/PVDF	34.0±0.6	20.3	487.9±0.3	499.5±0.9

DSC and DMA analysis of oven and OoO cured CFRPs

DSC and DMA analysis were utilised to evaluate the thermal and thermooechanical performance of both oven and OoO cured CFRPs, with the results summarized in Fig. S2. Glass transition temperature (T_g) were obtained from both DSC and DMA, using the midpoint type half height method and peak temperature of the tan δ curve respectively, as summarized in Table S2. Regarding the mechanical properties obtained from DMA tests, the oven and OoO cured composites showed similar storage modulus (E') at room temperatures, which were around 48 GPa and 50 GPa respectively.

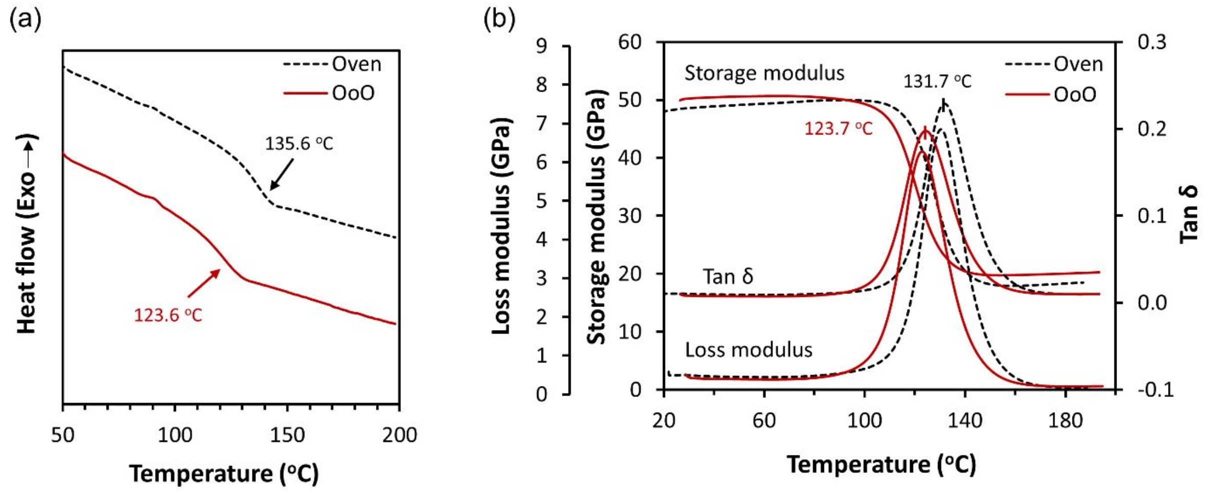

Fig. S2. Representative (a) DSC and (b) DMA results of oven and OoO cured CFRPs, with the glass transition temperatures, storage and loss modulus illustrated.

Table S2. Glass transition temperature (T_g) of oven and OoO cured CFRPs obtained from DSC and DMA.

	$T_{g,\ DSC}$ (°C)	$T_{g,\ DMA}$ (°C)
Oven	135.6±0.3	131.7±0.2
OoO	123.6±2.4	123.7±3.1

Fibre volume fraction of the cured CFRPs

Fibre volume fractions (V_f) of the panels were calculated based on the following equation:

$$V_f = W_f \frac{\rho_c}{\rho_f} \tag{5}$$

Where W_f is the fibre weight fraction which can be obtained via weighting the dry fibres and cured panels; ρ_c and ρ_f are the density of the composite panel (m_c/V_c) and fibre (1.78 g/cm^3). The average V_f of oven and OoO cured CFRPs are at 45.0% and 46.6% respectively.

References

1. Gupta TK, Choosri M, Varadarajan KM, Kumar S. Self-sensing and mechanical performance of CNT/GNP/UHMWPE biocompatible nanocomposites. J Mater Sci. 2018;53(11):7939–52.
2. Kashi S, Gupta RK, Kao N, Hadigheh SA, Bhattacharya SN. Influence of graphene nanoplatelet incorporation and dispersion state on thermal, mechanical and electrical properties of biodegradable matrices. J Mater Sci Technol. 2018;34(6):1026–34.
